# Considering the “Dog” in Dog–Human Interaction

**DOI:** 10.3389/fvets.2021.642821

**Published:** 2021-05-05

**Authors:** Alexandra Horowitz

**Affiliations:** Department of Psychology, Barnard College, New York, NY, United States

**Keywords:** human-animal interaction, dog-human interaction, welfare, animal use, experience, point of view, consent

## Highlights

- A focus on the experience of the silent partner in dog–human interaction research: the dog.
- Developing a vocabulary to discuss not just the welfare of the dog but also their perspective and agency.
- Raising issues about use of non-human partners, for the species, and for individual members of that species.

## Introduction

The lives of the contemporary human animal and other non-human animals are surprisingly antithetical. While one might imagine that our mutual membership in the animal kingdom would predicate reciprocal interactions, we instead have a largely imbalanced relationship with non-human animals (hereinafter, “animals”), with animals bearing the brunt of this imbalance. People eat animals for nourishment or enjoyment, keep animals captive for meat or as pets, cage animals for amusement, use animals as models for studying human disorder and disease, and kill animals for sport, for being a nuisance, or for being inconvenient. Even the research fields of animal behavior and animal cognition are not entirely exempt from this imbalance. Animal cognition, borne of comparative psychology, largely studies animals to determine how they reflect on human cognition; animal behavior research studies animals for their own sake, but often that research involves interfering with, maiming, or killing the animal in the course of research. Some widespread human behavior, such as keeping animals as pets in the home, does evince an interest in other animals, but it is worth remembering that this is a model of animal captivity, which also produces millions of homeless or unmanageable animals who are killed annually in the United States alone (1).

In this context, the field of human–animal interaction (HAI), which avows an interest in the salutary effect of interacting with animals, seems an anomaly. On examination, though, it appears to be another example of the antithetical approach that typifies our other engagements with animals. In all cases, animals are *used* by humans. In HAI research, the animal is a quiet partner, useful only for the effect their presence has on the person, and rarely considered in and of themselves. Such research is likely performed in large part by individuals who deeply care about animals, human or not; as a result, they may be able to take the lead in imagining how the non-human animal could also become the subject. In this opinion piece, I highlight the consideration of animals in HAI research and suggest some ways to foreground the animals so used.

While HAI is defined broadly to include myriad forms of interaction between the human and non-human animal, I will focus on dog-human interaction research as exemplary of HAI research in its breadth and aims. The great preponderance of this work investigates the effects on people of various characterizations (elderly, children, developmentally different) of various interventions or interactions with dogs. Dogs are a convenient species to work with, as they have long been domesticated: bred to feature traits and behaviors that appeal to us, such as their friendliness, adaptability to interspecific living, and attention to our attention (2, 3). Dogs are tractable, easily trained, and widely available. Many dog–human interaction studies investigate the interactions between dogs and their present owners, obviating the researchers' needs even to house or train animals.

Most dog–human interaction studies investigate whether a specified exposure to a dog is salutary to humans (4). The common-sensical notion that animals who are already inside our homes “must” be good for human health implicitly or explicitly drives this research. Research has looked at mental health (such as reducing stress), physical health (such as decreasing rates of asthma, obesity, and lowering blood pressure), and general socioemotional benefits [e.g., McCardle et al. (5)]. However, there is no unanimous consensus about the benefits of therapies for humans involving animals; results are equivocal [(6, 7); for a review of the kinds of results over the last decade, see Griffin et al. (8)].

In contrast to the myriad forms and number of studies on the effect for humans on the interaction, very few studies, relatively, gauge the effect—either short- or long-term—on the dogs involved (9–11). As of 2017, Glenk found just nine HAI studies in peer-reviewed journals considering the effect of the work on the dog. These studies attempted to measure the dog's welfare when participating in therapeutic situations known as animal-assisted interventions, animal-assisted therapy, or animal-assisted activity. Search terms by Glenk (10) reveal several additional published journal articles in the 3 years since her publication. While these additional papers represent a small fraction of the research published in these years on HAI, the idea that dog welfare is integral to the programs is clearly spreading. Recent studies use different methods of characterizing welfare, from physiological measures like heart rate and cortisol levels (12–17), to behavioral measures of stress, like panting, lip licking, and yawning (14, 16–18), which may partially explain why there are, overall, mixed results.

Another possibility for the mixed results is the great differences in the dogs themselves. Considering all individual dogs, across breeds, age, sex, temperament, personal history, and health, as representative “dogs” is characteristic of this work as published. Their status is operationalized: dogs are treated less as subjects than as stimuli. They are typically not described as subjects or participants; they are thus, by default, objects. Who they are as individuals is rarely acknowledged in published work. Examining how the dogs are described in papers on HAI research, we get a sense of their negligible status. As Griffin et al. (8) note, most studies have no information on even very basic demographics of the dogs, such as sex, age, breed, desexing status, or training history. Even in the research designed to investigate the welfare of dogs in HAI work, who the dogs are is often underspecified. At best, sex, breed, age, health, living situation, weight, and source of dog, if known, is reported (19–21), although these figures may be averaged. In other work, neither individual breed nor sex information is given, nor is any life history (22, 23). A few papers with single subjects do better, such as Piva (24), which describes not only the typical demographics of the dog, but her personality with people, her testing temperament, her skill at performative obedience, and additional physical features.

This deficit is analogous to the report of animals kept—and the conditions in which they are kept—in most scientific work, historically. As Adams (25) notes about Ivan Pavlov's research, for instance, though it is widely cited, and clearly represented as involving dogs, no details of the dogs, such as the length of their lives, the conditions (social, living) of their lives, the procedures done to them, or even how their lives ended, are included either by Pavlov or by the textbook authors or journal papers that cite the research.

Only rarely are dogs named in the published reports of these studies [see Clark et al. (12), for a single instance]. The longstanding prohibition against naming animal subjects in behavioral science was famously flouted (if inadvertently) to great effect by Jane Goodall (26); since then, though animals might be named by researchers, they are still infrequently named in reports of the research results (whether researchers included the names in submitted manuscripts or not). Naming makes something someone: it personalizes them (27). To give an animal a name highlights the differences between subjects (individuals) being considered only as members of a group (species). In a postscript to his paper, Adams (25) lists the names of some of Pavlov's dogs, as a way to begin to remedy their oversight. By not naming dogs, researchers demonstrate that they are not considering dogs as individuals at all; they are simply thought of as representative “dogs.” It is perhaps no wonder, then, that their well-being is not being examined: only individuals can have well- or ill-being at all.

What do these observations about the status of dogs in HAI research highlight? Significantly, they highlight that our society supports animals being used—used for the sake of another: the human animal (4). Can using animals for our purposes be justified? One hundred years ago, it did not seem roundly exploitative to keep animals in cages for research by humans—for the sake of human health, curiosity, or anything else. As much as societal opinion about such uses has changed since that time, one wonders whether considering animals as only the material with which to look at our own health is similarly exploitative (or will look exploitative in another century). Even in HAI research (with dogs, but also with horses and other domestic animals) in which the humans involved consider the animals their “partners” or “companions,” the study subject is mostly one-sided.

“Use” need not necessarily mean “exploitation”: to not exploit, but merely use animals, one must make choices that further the welfare of the animal, even if it is in conflict with one's motives (28). This definition prompts the further question of whether the very process of domestication, as traditionally conceived of Clutton-Brock (29), and breeding—focally redesigning a species to suit our whims—might be seen as exploitative. There certainly have been deleterious results for many domesticated animals: they have become largely food products, their natural life cycle and their normal social behavior disrupted. While domestic animals kept as pets do, in some cases, enjoy freedom from many of the pressures of living independently of humans, and are often loved (whether the expression thereof is beneficial for them or not), they are constitutionally “captive” (30).^1^

While not expecting HAI researchers to solve the global question of animal use, within the field there is much room to mitigate the problems associated with use. I consider a few below: beginning to see, through identification and description, the animals involved in research; working toward positive welfare for animal participants; and appreciation and formal acknowledgment of the animal experience.

### Who Is the Dog?

While on its face it does not sound disparaging or incomplete, in HAI work, dogs are just “dogs.” What they are not are: subjects, agents, individuals, sentient participants. Not only their names, but basic facts about each dog's biology, behavior, and personalities are often completely absent from reports of research critically involving them. In considering animals in science, Birke (31) discusses a group of research animals seen as “numbers, as tools of the trade,” “whose experiences are considered unimportant”: she is referring to lab animals. We assume that the experience of dogs in HAI research far surpasses that of dogs living their lives in laboratories—but that's just the point: without the research, we can only assume. We will make mistakes about their experience if we do not even look. We need to begin to see the dogs in the research. Who are they? What are their histories; what are their preferences; what are their personalities? Indeed, it is because of their personalities that dogs are valued for much HAI work: so can we describe them? Use without identity promotes the ongoing inequity, the “moral discontinuity” that not only is one kind of life more valuable but also that only one kind of life deserves to be seen (25, 31).

### A Good Life

As seen above, there is an increasing volume of work aiming to identify markers of stress and anxiety in dogs in intervention and interaction settings. In other words, this research looks to identify whether there are any negative effects for dogs. Recently revised standards for work with dogs in animal assisted interventions lay out guidelines to ensure the “health, welfare, and well-being of dogs,” aimed to avoid poor welfare: for instance, that “least restrictive, minimally aversive” training methods are used (32). But the absence of poor welfare does not imply the presence of positive welfare (33). The increasing volume of work on the ethics of animal use and on animal welfare is apt; the next step is to determine what interactions *improve* animals' health and well-being—which are salutary and appropriate for the individual animal. Dogs' positive welfare should itself be a focus of investigation [as it is beginning to be in other contexts: for instance, 4 of the 22 behaviors looked at in a recent study on child–dog interactions are markers of positive welfare (34)].

Societally, in the last two centuries we have seen legislated concerns for animal well-being in the form of animal cruelty laws (in the US); notably, though, such laws only deal with truly gross disruptions of needs and well-being, such as killing or torturing [and even those are permitted if deemed “necessary” (27)]. Recent research has begun to address what animals not only need, but want (35–37); such standards should be applied not only to the most egregious cases of animal use, such as invasive laboratory experimentation, but to all animal uses.

Relatedly, currently best-practice recommendations for HAI research emphasize the importance of using animals who are appropriate and appropriately trained for the work; monitoring of their welfare during the work; and allowing for retirement of an animal from work (6, 32). At the same time, animals need to be “controllable” (38), to be polite, “not regularly vocalize inappropriately” (32), and to react (unnaturally) calmly to arousing stimuli. We could ask whether such work curtails an animal's full expression of a natural life (39), flourishing at whatever “sort of thing” the animal is (40): the dog's capacity for dogness (27). The biological needs and desires of non-humans are not identical to human needs (41), so advancing their welfare requires an understanding of the dog's perspective—an understanding that has hardly begun to be pursued in any field.

To begin, we can imagine that a good life for dogs includes not only freedom from suffering and establishment of general bodily integrity and well-being, but stimulation of the senses, an ability to run around; chances to do new things or familiar activities; dog and person companionship and physical interaction; to engage with the natural world, sniffing and rolling; and to have some control over their days and environment. Opportunities for play, joy, amusement, attachment, choice, exploration, and periods of rest are all salient. There is moral work yet to be done to ensure that the animals' health and well-being is prioritized.

### The Dog's Point of View

Imagining the lived reality of a working or therapy dog's experience is critical to an understanding of what their needs might be. Like most owned companion dogs, dogs used in therapy have few choices but to go along with their owner or handler. While proper training and selection for dogs used in therapy settings usefully exposes them to, broadly, unfamiliar and various social and sensory situations, our imagination about the dogs' experience may limit our ability to anticipate their experience in a working setting. For instance, human unfamiliarity with the dog's strongly olfactory rendering of the world means that few attempts are made to predict or account for new smells associated with new settings and people. Odors are not experienced at the same rate as light is: smells emit from sources but need to be closely examined or to travel on air currents in order to be perceived, unlike seen objects, which just “appear” at once before our eyes, if there is no obstruction (3). Thus, the pace at which one might explore—and “see”—a new olfactory environment might be different than a new visual environment. Sound lands differently on dog ears than on human ears, given their proximity to the reflective surface of the ground; moreover, they are sensitive to higher frequencies than our ears can detect, enabling perception of ultrasonic sounds produced by rodents or insects in the environment (42); vocalizations of children, often shrill, are less anticipatable by a dog than by a human conspecific (38). Dogs are often used exactly to be touched; while dogs vary in their endurance of stressful touching, such as hugs or head pats, even in the best cases this is a demand on the dog (3, 38).

### A Modest Proposal: Asking for Consent

Perhaps most fundamental to considering dogs in HAI research is a clear delineation of the role of the dogs. If they are subjects, they should be thoroughly described, and the effect of the work not only in the short term, but also in the long term, should be investigated. Moreover, more work should be *designed* to specifically gauge their welfare, rather than assessing it as an afterthought. Welfare should include not only a lack of negative effects, such as an increase in stress levels, but also an increase in positive effects. While most work on the welfare of dogs involved in HAI research does the former, almost none does the latter.

By neither considering welfare assessment as integral to research, or even describing who the dogs are, most HAI research is using dogs as objects only. We can question whether this is justifiable with a sentient animal. Ought dogs, or any animal, be used to attempt to improve the lives of humans? One possibility might be to continue to allow use, but insist on consent—consent of the dogs to their participation. As sentient animals, dogs are, whether bred for work or not, experiencing their lives. They have preferences and emotions. Despite their selection for compatibility with humans, dogs are likely to show stress behaviors—as many handlers are already seeing (43). Use of dogs might be permitted if they are able to “opt in” and “opt out” of being so used, just as a human participant in research can give or withdraw consent. Insofar as the research examines the human–animal bond, voluntary participation is an essential element of a bond-like relationship (44).

Determining consent is not as tricky as it might seem with non-language-using animals. Many dog trainers have encouraged straightforward consent training, wherein dogs are taught behaviors they can employ to agree to participation in a medical procedure, for instance. Having a choice is a way to grant animals agency, central to good welfare (45). Additionally, human handlers or experimenters can be better trained to read body language of dogs that indicates that a dog agrees to participation, is simply enduring participation, or would rather not participate. Meints et al. (46), for instance, recently demonstrated not only how erroneous peoples' reading of dog behavior is, but how readily people can learn to correctly interpret dog signals. Still, validated standards of consent would lift this requirement out of individuals' judgments to a societal level. Just as human participants must give consent for participation for research to be conducted and published, having animal participants consent could eventually be required for publication of research involving them.

## Conclusion

Ultimately, though joined to us by a hyphen, the animal in the “human–animal” interaction is largely neglected in published research. With a contemporary, and scientifically validated, understanding of animals such as dogs as sentient, their role must be seriously considered. In particular, I recommend that researchers and handlers be mindful of the animals' perspectives of the activities they are engaging in; strive not just for lack of poor welfare but also the presence of positive welfare; and work toward standards of affirmative consent. Some of this work could be aided by publishing concerns, which typically require summary information about animals: journal editors might, instead, emphasize the importance of specific information about individual participants, as well as encouraging consent. Today, dogs are too often operationalized in HAI research, rather than seen as individuals with experiences—whose experience ought to be foregrounded.

## Author Contributions

AH is the sole contributor to this paper.

## Conflict of Interest

The author declares that the research was conducted in the absence of any commercial or financial relationships that could be construed as a potential conflict of interest.

## Footnotes

^1^A current debate asks whether dogs were exclusively domesticated by humans or whether wolves essentially “self-domesticated” into proto-dogs, insofar as there may have been self-selection by ancient wolves before selective breeding by humans began thousands of years ago. In either event, the result is that dogs are considered “domesticated,” and the process of human selection is several 1000 years old.
